# Advantages of intensity modulated radiotherapy in recurrent T1-2 nasopharyngeal carcinoma: a retrospective study

**DOI:** 10.1186/1471-2407-14-797

**Published:** 2014-11-03

**Authors:** Sufang Qiu, Jun Lu, Wei Zheng, Luying Xu, Shaojun Lin, Chaobin Huang, Yuanji Xu, Lingling Huang, Jianji Pan

**Affiliations:** Department of Radiation Oncology, Fujian Provincial Cancer Hospital, Provincial Clinical College of Fujian Medical University, Fuzhou, Fujian People’s Republic of China; Fujian Provincial Key Laboratory of Translational Cancer Medicine, Fuzhou, Fujian People’s Republic of China; The Teaching Hospital of Fujian Health College, Fuzhou, Fujian People’s Republic of China

**Keywords:** IMRT, Recurrent T1-2 nasopharyngeal carcinoma, Re-irradiation treatment

## Abstract

**Background:**

Recurrent T1-2 Nasopharyngeal Carcinoma (rT1-2) may be salvaged by 3D – CRT (3D-Conformal Radiotherapy), IMRT (Intensity Modulated Radiotherapy), Brachytherapy (BT), BT with external radiotherapy. The purpose of this study is to address the efficacy and toxicity profile of aforementioned four modalities for rT1-2 NPC.

**Methods:**

168 patients, median age 48 years (range 16–75 years) proven rT1-2 NPC were diagnosed and treated with four different irradiation modalities (3D-CRT, IMRT, BT, BT with external radiotherapy). Median time to recurrence was 30 months (range 1–180 months). The median follow-up time was 28 months (range, 4–135 months).

**Results:**

161 patients completed a median dose of 6445 cGy (ranging 30 to 87 Gy). Seven patients prematurely terminated their treatment due to acute side-effects and received 30–49 Gy. The 1- and 3-year local regional recurrent free survival (LRRFS), distant free survival (DFS), and overall survival (OS) rates were 82.03% vs. 82.03% vs. 82.58%, 51.33% vs. 51.33% vs. 53.41, respectively. Gender and recurrence T-classification were the two significant adverse prognostic factors for LRRFS, DFS, and OS rates. Grade 3 or 4 toxicities were tolerable.

**Conclusion:**

3D-CRT, IMRT, BT, BT with external radiotherapy are feasible and efficacious for rT1-2 NPC. In toxicity 3D-CRT/IMRT group is lower than BT group. IMRT is superior for rT1-2 NPC.

## Background

Nasopharyngeal carcinoma (NPC) is considered an endemic carcinoma in Southern China. Fujian province is one of the high incidence regions for NPC [1]. It is a radiosensitive disease and radiation therapy is the mainstay treatment of non-metastatic NPC. The 5-year OS rate ranges from 75-82% for NPC patients. The local recurrence-free survival rate exceeds 90% [2]. Despite the high efficacy in locoregional disease control with high-dose radiation, local recurrence remains a major cause of treatment failure for T1-2. However, treatment of NPC recurrence, even in early T stage, poses a challenge [3].

Various strategies, including surgery [4] (i.e., nasopharyngectomy), brachytherapy (BT) [5], stereotactic radiosurgery [6] and external radiation [7, 8], have been used in an attempt to cure local early recurrent NPC. Considering the nasopharynx structure, small tumors may be difficult to access. In addition, high dose re-irradiation will have extensive side effects. Therefore, only a few patients accept nasopharyngectomy or stereotactic radiosurgery. Re-irradiation remains an important modality for re-treatment. 3D-Conformal Radiotherapy (3D-CRT) [6]. Intensity Modulated Radiotherapy (IMRT) [6, 9] and brachytherapy (BT), are often utilized for nasopharynx local small lesions, and treatment enables the delivery of high-dose radiation to the target volume(s) while protecting normal radio-sensitive normal tissue and organs.

However, the available literature comparing the disease control and treatment-induced side effects from re-irradiation modalities in rT1-2 NPC is scant [10].

The aim of this study is (1) to document the outcome for 4 re-irradiation treatment modalities for rT1-2 NPC treated with 3D-CRT, IMRT, BT, and BT with external radiation, (2) to assess efficacy and late toxicities and (3) to determine which one is the best treatment method. We have large sample of 168 cases of rT1-2 NPC, all from Fujian Provincial Cancer Hospital, with strong homogeneity between 1996 and 2009.

## Methods

### Patients and pretreatment evaluation

Between January 1996 and June 2009, a total of 168 patients (median age 48 years, range 16–75 years) with histological proven local (rT1-2) NPC were diagnosed and treated with four different irradiation modalities (3D-CRT, IMRT, BT, and BT with external radiotherapy). Pretreatment evaluation includes electrocardiogram, urinalysis, disease history, bone scan, routine examination, blood counts, serum electrolytes, chest X-ray, fiberoptic nasopharyngoscopy, head and neck CT scan, and ultrasound or CT of the abdomen. In addition, magnetic resonance imaging (MRI) scans of the head and neck were applied instead of CT in all patients after July 2005. Other examinations and studies such as position emission tomography (PET) scans were performed at the treating physician’s discretion. NPC recurrence was histologically confirmed in all cases through biopsy of the recurrent foci at the posterior nasal space. Biopsy of the neck adenopathy was not performed for the ten patients who presented with regional recurrence. All cases were restaged according to the American Joint Cancer Committee (AJCC) 1997 staging classification. The characteristics of the 168 patients are detailed in Tables 1 and 2.

**Table 1:** **Baseline characteristics of cohort**

	Number	%
Age, year		
≥ 50	71	42.3%
<50	97	57.7%
Gender		
Male	136	81%
Female	32	19%
T-Classification		
1	10	6.0%
2	86	51.2%
3	46	27.4%
4	26	15.5%
rT-Classification		
1	65	38.7%
2	103	61.3%
Time to recurrence (months)		
1-11	28	16.7%
12-23	37	22.0%
24-35	45	26.8%
36-59	29	17.3%
>60	29	17.3%
Median = 30 months		
Treatment		
3D-CRT	67	39.9%
IMRT	28	16.7%
Brachy	20	11.9%
Brachy + ER	53	31.5%
Dose (Gy)		
<60	39	23.2%
≥ 60	129	76.8%

*Abbreviation*: *ER* external radiation, *3D-CRT* three-dimensional conformal radiotherapy, *IMRT* intensity modulated radiotherapy, *Brachy* Brachytherapy.

**Table 2:** **The patient distribution number of 3D-CRT, IMRT and BT +/− ER group**

T	3D-CRT	IMRT	BT +/− ER	X ^2^	***P***value
1 + 2	32	17	47	4.117	0.128
3 + 4	35	11	26		

*Abbreviation*: *BT +/− ER* brachytherapy +/− external radiotherapy.

### Ethics

The research had been performed with the approval of Fujian Cancer Hospital Ethics Committee of Fujian Medical University. The reference number is FJCH-09911. Written informed consent was obtained from each patient. If the patients were children, written informed consent was obtained from their guardians.

### Irradiation therapy

All patients (3D-CRT, IMRT) were fixed in the supine position with thermoplastic masks. CT simulations with intravenous contrast using 3 mm cuts from the vertex to 2 cm below the clavicular heads were performed. MRI-CT fusions using the Oncentra Masterplan co-registration software (Oncentra Masterplan® version 1.5, Nucletron BV) were conducted for all cases treated after July 2005.

The gross tumor volume on the primary site and neck (GTV-P and GTV-N, respectively) included all disease visualized either on CT or MRI or both CT and MRI. Clinical target volumes (CTVs) of both GTV-P and GTV-N included microscopic disease by adding up to 8–10 mm to GTVs. However, smaller margins around 3 mm were allowed when CTVs are near critical organs, such as the brain stem or the spinal cord. High-risk areas, such as draining lymphatics were also prevented if possible. An additional 3 mm margin was extended to CTVs to create the planning target volume (PTV) to allow for a setup variability and internal motion.

**Figure 1:**
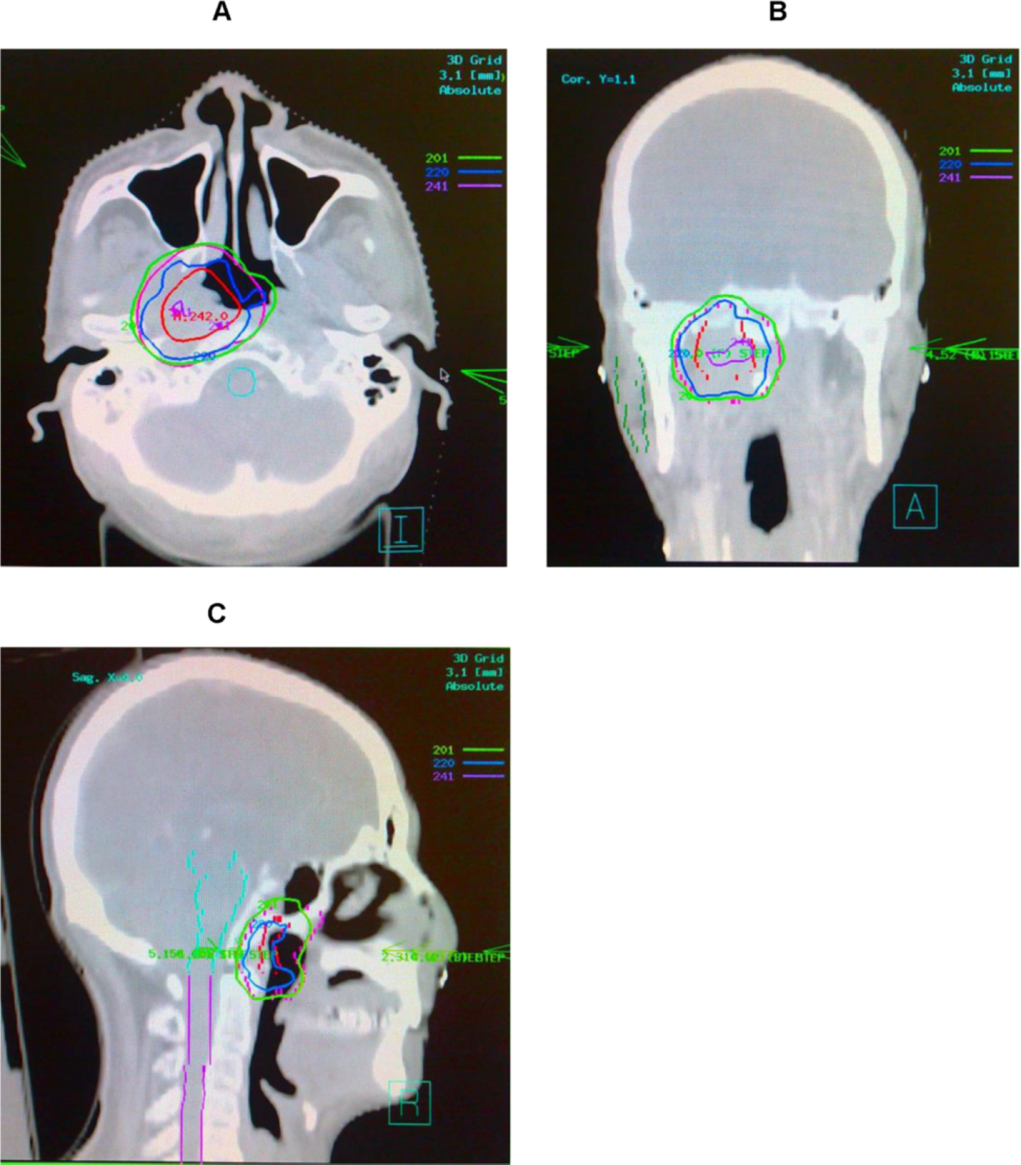
**CT simulation images of a patient with rT2N0M0 NPC. A**: Transverse CT simulation images at the levels of superior levels of the nasopharynx illustrating target volumes, normal structures and isodose lines showing doses per fraction. **B** and **C**: Coronal (B) and Sagittal (C) CT simulation images illustrating target volumes, normal structures and isodose lines showing doses per fraction.

**Figure 2:**
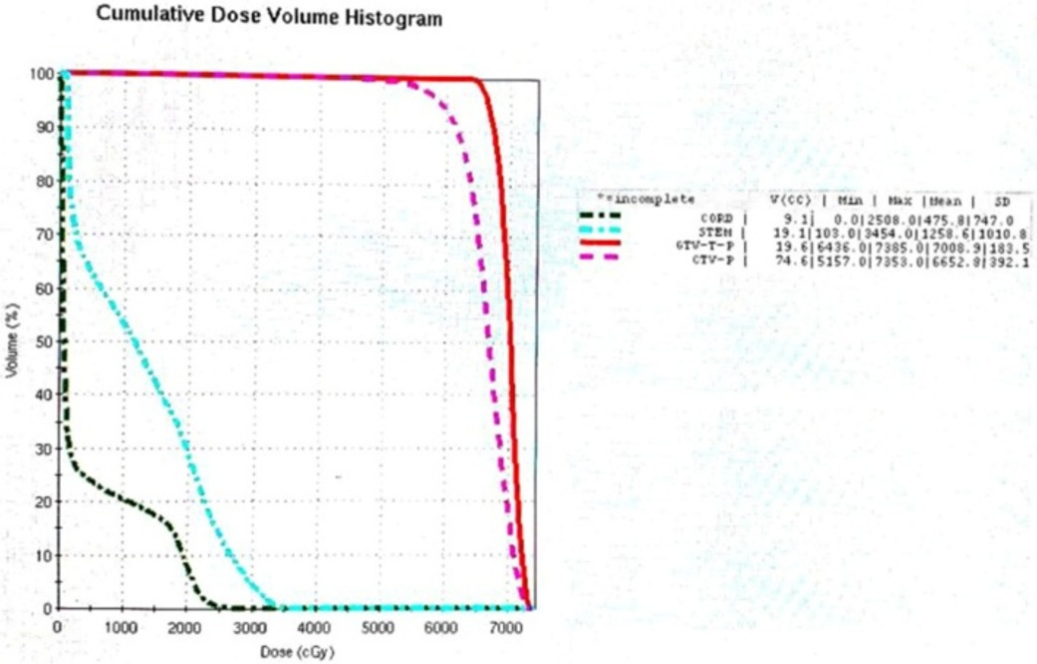
**Dose-volume histogram of the same patient.**

Endanger normal structures including the optic nerves and chiasm, brainstem, spinal cord, temporal lobes, eyeballs and lens, pituitary gland, temporomandibular joints (TMJ), as well as parotid glands were delineated and described as organs at risk (OARs) during planning. Total dose to the spinal cord, brainstem, and temporal lobes, optic nerve/chiasm, TMJ, eyeballs and lens were required to be measured during planning and their limitation was individualized based on doses delivered from the primary radiation therapy. Inverse treatment planning using the Plato® treatment planning software system (RTS® version 2.6.4) and a mono-isocentric technique was used for every patient in this cohort. The iso-center was set at the center of the GTV-P. Minimal planned doses between 50 and 60 Gy (2 Gy or 1.8 Gy per daily fraction, five days per week) were prescribed to the PTV(s) for all patients. The PTV(s) were treated with step-and-shoot IMRT using 5–7 coplanar beams, using a computer-controlled auto sequence multi-leaf collimator (MLC) on a linear accelerator (Elekta Precise®, Elekta AB) contained with a 40-leaf MLC. A treatment plan of a patient with local recurrence only is illustrated in Figures 1 and 2.

The hyperfraction radiation refers to treatment twice or more than twice every day, with an interval of at least six hours, each fraction lower than the routine dose, compared with the conventional dose. It has similar or higher total tumor radiation dose and aims to decrease toxicities and increase the tumor control rate. Our center was the first in China to adopt twice daily fractions, with intervals of six hours between the two fractions with the aim of reducing late damage.

### Follow-up

All patients were followed up on a weekly basis during their treatments. Then, they met their attending physicians three months in the first two years and 6 months for additional three to five years, and annually after five years according to our therapeutic protocols. A complete examination, as previous described, was requested at each follow-up as well. Meanwhile, side effects to treatment were evaluated according to the RTOG/EORTC radiation morbidity scoring criteria at each follow-up according to Cox [11].

### Statistics

The local regional recurrence-free survival (LRRFS), disease-free survival (DFS), and overall survival (OS) rates were estimated with the Kaplan-Meier method. Log-rank tests were performed to detect differences in survival among different prognosticators. Multivariate analysis using the Cox proportional hazard model was performed for all prognostic factors. Level of significance was set at a 2-tailed P value of <0.05. All analyses were conducted using the Statistical Package for the Social Science (SPSS) software, version 17.0 (SPSS, Chicago, USA).

## Results

### Treatment outcome

The median follow-up time for the entire group was 28 months (range, 4–135 months). One hundred and sixty-one patients completed their planned radiation to a median dose of 6445 cGy. Seven patients prematurely terminated their treatment due to acute side-effects and received doses between 30-49 Gy. The 1-, 3-, and 5-year LRRFS were 82.03% vs. 82.03% vs. 82.58%, DFS 51.33% vs. 51.33% vs. 53.41, and OS 35.52% vs. 34.85% vs. 37.99%, respectively. At the time of this analysis, 92 (54.8%) were deceased; 37 due to progressive/recurrent local diseases, 26 due to distant metastasis, 5 due to secondary primary malignancies, 3 due to an accident, 10 because of excessive nasal bleeding, and 8 due to other medical conditions. Unfortunately, there were three death cases with no detail medical records. Of the remaining 76 surviving patients at the time of censorship, 6 had local recurrence after re-irradiation and one developed distant bone metastasis.

### Prognostic factors

These prognostic factors, including age, gender, and T-Classification at the initial diagnosis and recurrence, time to recurrence, the dose, and the modalities of re-irradiation on predicting local control (LC), DFS, and OS were evaluated by both univariate and multivariate analyses. Gender and recurrence T-classification were the two significant adverse prognostic factors for LC, DFS, and OS rates in both univariate and multivariate analyses, whereas the modalities of re-irradiation including four salvage radiotherapy techniques were not statistically different for LC, DFS, and OS rates (Tables 3 and 4).

**Table 3:** **Univariate analysis of potential prognostic factors**

		OS		DFS		LC	
Item	n	Mean survival time ± SE	***P***value	Mean survival time ± SE	***P***value	Mean survival time ± SE	***P***value
Gender			0.013		0.018		0.015
Male	136	65 ± 5		61 ± 5		62 ± 5	
Female	32	40 ± 7		38 ± 6		38 ± 6	
Age (y)			0.858		0.868		0.779
<50	93	58 ± 6		55 ± 5		55 ± 5	
≥50	75	60 ± 7		57 ± 6		58 ± 7	
T- classification			0.795		0.647		0.720
1 + 2	96	68 ± 9		54 ± 5		55 ± 5	
3 + 4	72	56 ± 5		62 ± 7		62 ± 7	
rT- classification			0.057		0.232		0.186
1	65	65 ± 7		58 ± 6		60 ± 6	
2	103	54 ± 5		53 ± 5		53 ± 5	
Time to recurrence			0.38		0.293		0.334
<36 months	65	66 ± 7		64 ± 7		64 ± 7	
≥36 months	103	55 ± 5		51 ± 5		52 ± 5	
Treatment			0.707		0.749		0.715
3D-CRT	67	56 ± 7		56 ± 7		56 ± 7	
IMRT	28	55 ± 7		55 ± 7		55 ± 7	
Brachy	20	59 ± 9		47 ± 8		47 ± 8	
Brachy + ER	53	60 ± 7		57 ± 7		58 ± 7	
Dose (Gy)			0.304		0.221		0.246
<60	48	68 ± 9		66 ± 9		66 ± 9	
≥60	120	56 ± 5		52 ± 5		53 ± 5

**Table 4:** **Multivariate analysis of potential prognostic factors**

Endpoint	Factors	B	SE	WALD	Df	Sig	Exp(B)	95%CI EXP(B) Lower upper	
OS	Gender	−0.74	0.258	8.312	1	0.004	0.475	0.286	0.788
	rT- classification	0.682	0.274	6.208	1	0.013	1.978	1.157	3.382
DFS	Gender	−0.67	0.25	7.135	1	0.008	0.511	0.312	0.836
	rT- classification	0.58	0.26	4.948	1	0.026	1.786	1.071	2.977
LRRFS	Gender	−0.70	0.25	7.764	1	0.005	0.496	0.303	0.812
	Rt- classification	0.619	0.26	5.536	1	0.019	1.857	1.109	3.11

*Abbreviations*: *OS* overall survival, *DFS* disease-free survival, *LRRFS* locoregional recurrence free survival.

### Toxicities

All patients except 7 tolerated their re-irradiation well and completed the planned therapy. The seven patients terminated their treatment between 30 Gy and 49 Gy due to acute mucositis. Severe adverse effects (defined as Grade 3 or 4 toxicities described by the RTOG/EORTC late toxicity criteria) were observed after 3 months following the completion of re-irradiation and included: 23 patients (13.7%) with ulceration in the posterior nasal space, 29 patients (17.3%) with cranial nerve palsy, 22 patients (13.1%) with trismus, and 27patients (16.1%) with hearing deficit [11]. As all patients presented with xerostomia after their primary radiation therapy, severity and frequency of xerostomia was not recorded and analyzed (Table 5). We divided the cohort toxicity into three groups; 3D-CRT, IMRT and brachytherapy and/or external radiation (BT +/− ER). We found the nasopharyngeal ulcer, cranial nerve palsy and hearing deficit is a significant difference among the three groups; the 3D-CRT and IMRT group had a lower incidence than the BT +/− ER group. The trismus was similar in the three groups. IMRT group had a lower incidence than 3D-CRT group in the toxicity, but the difference was not statistically significant [11].

**Table 5:** **T comparison of late radiation complications on three groups**

Complication	Radiation technique			***P***value
	3D-CRT (n = 67)	IMRT (n = 28)	BT +/− ER (n = 73)	
Nasopharyngeal necrosis	4 (6.0%)	1 (3.6%)	18 (24.7%)	0.001
Cranial nerve palsy	7 (10.4%)	2 (7.1%)	20 (27.4%)	0.008
Trismus	9 (13.4%)	4 (14.3%)	9 (12.3%)	0.961
Hearing deficit	6 (9.0%)	2 (7.1%)	19 (26.0%)	0.008

## Discussion

Fujian province of Southern China is a high incidence region for NPC. The 168 cases of recurrent T1-2 NPC were all from Fujian Provincial Tumor Hospital between 1996 and 2009 and had strong homogeneity. In this series of 168 patients diagnosed with locally recurrent T1-2 NPC and previously treated with a definitive dose of radiation, high-dose re-irradiation with 3D-CRT, IMRT, BT, and BT with external radiotherapy is feasible and efficacious. The estimated LRRFS, DFS and OS rates at 1-, 3-, and 5 years were 82.03%, 82.03%, and 82.58%, 51.33%, 51.33%, and 53.41%, 35.52%, 34.85%, and 37.99%, respectively. Multivariate analyses revealed that gender and recurrent T-classification were two significant prognosticators for both LC and OS after re-irradiation. Additionally, most patients tolerated their retreatment, although a significant minority still suffered at least one moderate to severe late radiation toxicity.

Local recurrence of NPC in the post-nasal space and base of skull poses a major challenge for treatment; nevertheless, retrospective evidence from a large series suggests that salvage treatment for isolated local recurrences may improve survival, especially for small (rT1-2) volume recurrent disease [3]. For tumors localized to the nasopharynx, surgery or brachytherapy may be viable options. Good tumor control with acceptable morbidities has been reported with salvage nasopharyngectomies performed in expert centers, intra-cavitary or interstitial brachytherapy are alternative modalities for limited local recurrences [12]. Law et al. recently published their series of intra-cavitary mold brachytherapy with 50-55 Gy using a 192Ir source, and demonstrated a 5-year local control rate of 85% and a major complication rate of 47% [13]. In addition, Leung et al. described salvage therapy with a combination of high-dose-rate (HDR) intra-cavitary brachytherapy and external beam radiation therapy, and found that a higher radiation dose and a smaller recurrence was associated with improved outcomes [14]. Stereotactic radiosurgery or radiotherapy have also been employed to treat locally recurrent NPC [15]. This highly precise technique allows a delivery of ablative radiation doses with a rapid fall-off and is well suited to the clinical situation where critical structures lie in proximity to the posterior nasal space. Additionally, where the tumor can be well visualized with fusion MRI/CT imaging and sufficient immobilization can be achieved is excellent for using customizable framed or frameless solutions. Multiple series have shown good efficacy, with 2-year local control rates ranging from 55% to 92%. However, morbidity after radiosurgery can be considerable, which may include carotid or cerebral hemorrhage, cranial neuropathy, massive epistaxis, nasopharyngeal necrosis, temporal lobe necrosis, and osteoradionecrosis of the skull base. Some of these severe toxicities may be related to the large fraction size used in previously heavily irradiated normal tissues [16].

All of the above-mentioned techniques may be applied for selected cases, especially for smaller volume recurrences within the nasopharynx within specialist centers. 3D-CRT and IMRT are advanced techniques that enable the delivery of satisfactory high-dose radiation to the target volume(s) while defending normal OARs. Consequently, they potentially improve the radiotherapy effect [17]. The clinical superiority of IMRT as a primary treatment technique with respect to both disease control and side effects has been repeatedly proved for newly diagnosed NPC [18–20]. The literature indicates IMRT is superior to 3D-CRT in planning the target and late toxicity [17, 21]. Re-irradiation of NPC local recurrence using IMRT is a relatively new concept and has been documented in 3 preliminary reports. The initial experience of 49 patients with recurrent NPC reported by Lu et al. [22] indicated that a sufficient coverage of tumor volume could be achieved using IMRT. Locoregional control rate of 100% was observed at the average dose of 71.4 Gy to GTV and a median follow-up time of nine months, Another previous article mentioned 31 patients with locally recurrent NPC treated with IMRT to a median dose of 54 Gy resulted in one-year locoregional progression-free and OS rates of 56% and 63%, respectively, after a median follow-up of 11 month [23]. In our previous paper, 70 patients were proven locally recurrent NPC with radiologic or pathologically when cured with IMRT [24]. The median time to recurrence was 30 months. Fifty-seven percent of the patients were classified as rT3-4. The minimum planned doses were 59.4 to 60 Gy in 1.8 Gy to 2 Gy fractions to the gross disease, with or without chemotherapy. The median dose to the tumor was 70 Gy (range, 50–77.4 Gy). With a median follow-up time of 25 months, the 2-year LRRFS, DFS, and OS rates were 65.8%, 65.8%, and 67.4%, respectively. Moderate to severe late side effects were noted in 25 patients (35.7%). Extended disease-free interval and advanced T classification at presentation were adverse prognostic factors. Han et al. investigated 239 local recurrence NPC patients with IMRT and claimed 5-year local recurrence-free survival (LRFS), distant metastasis-free survival (DMFS), DFS and OS rates were 85.8%, 80.6%, 45.4%, and 44.9% respectively [9]. All researchers have concluded that retreatment using IMRT is feasible and tolerated for patients who have experienced a definitive dose of radiation using a conventional technique for their primary treatment of NPC. In our study, there is no statistically significant difference among the four retreatment modalities for LC, DFS, and OS rates, because the four methods are all accurate radiotherapy, T1-2 patients recurrent target area tumors are small and easy to treat [17]. We may not detect a better effect of IMRT because the number of patients receiving IMRT is only 28, and we need more IMRT patients to confirm the results.

Our results indicated gender and recurrent T classification alone are not good prognostic factors. Males have a better prognosis than female because males have better tolerance of radiation than females. Our study shows that the extent of recurrent disease (i.e., rT-classification) was significant for predicting treatment outcome. The rT-classification of our patients was defined using the AJCC system for NPC, which is largely designed for initial staging. There are many published studies that indicate the earlier T classification of recurrent tumors, the better the prognosis [25–27]. We find that the toxicity that manifests as nasopharyngeal ulcer and cranial nerve palsy is significantly different between the BT group and non-BT group (3D-CRT, IMRT group). The toxicity in the non-BT group is lower than in the BT group. The trismus is similar in both groups. So we prefer to use 3D-CRT or IMRT rather than brachytherapy in rT1-2 NPC [25]. Chen et al. shows late toxicities are higher in a three-dimensional conformal group than in an intensity-modulated radiotherapies group for nasopharyngeal carcinoma [10]. In our study IMRT group had a lower incidence than 3D-CRT group in the toxicity, but the difference was not statistically significant. Because the number of patients receiving IMRT is only 28, we need more IMRT patients to confirm the results. Considering the balance of efficacy and toxicities, we think IMRT is the best choice for rT1-2 nasopharyngeal carcinoma.

Despite the relatively large sample size of this group of patients with locally recurrent T1-2 NPC, a number of pitfalls need to be discussed. The follow-up time of 28 months is relatively short for long-term outcome in head and neck cancer management. Nevertheless, in most cases, local recurrences of nasopharyngeal cancer occur in the first two years after IMRT treatment [26]. In fact, our observation period with a median follow-up time of 28 months may be adequate. Furthermore, this retrospective series may be with analysis inherent biases in nature.

Our results are far from conclusive and a number of critical questions need to be answered. Tian et al. [26] described a retrospective series of 251 patients with IMRT of locally recurrent NPC. The mean dose to the GTV was 70.04 Gy (61.73-77.54 Gy), but the re-irradiation dose is not prognostic factors. Chen et al. [28] demonstrated that IMRT with 70 Gy was efficient for local tumor control. However, they observed a high frequency of serious late complications. In our results, the prognosis of the high dose group is not better than that of a low-dose group. So how much is the best reasonable dose, and whether patients with recurrence needed high dose radiation is controversial. Clearly the optimal dose for disease control in re-irradiation for locally recurrent NPC needs to be determined.

## Conclusion

Four modes of re-irradiation treatment (3D -CRT, IMRT, BT, and BT with external radiotherapy) are feasible and efficacious for recurrent T1-2 Nasopharyngeal carcinoma. For toxicity, the 3D-CRT/IMRT group is lower than the BT group. IMRT is superior for recurrent T1-2 nasopharyngeal carcinoma.
